# Accuracy of dentalmonitoring’s artificial intelligence in detecting aligner tracking issues: a retrospective multi-centric study

**DOI:** 10.1186/s12903-025-07580-0

**Published:** 2026-01-07

**Authors:** Julie Fahl McCray, William Dabney, Dylan Handlin, Logan Smith, Jessie Zhu, Maria Roxas Regina Trinidad, Naurine Shah, Sarah Zaki, Rayan Skafi, Mohammed H. Elnagar

**Affiliations:** 1 Department of orthodontics, Center of Advanced Dental Education, Saint-Louis University, 3320 Rutger St., St. Louis, MO 63104 USA; 2 304 Browns Hill Court, Midlothian, VA 23114 USA; 3 Department of orthodontics, University of Alabama, School Of Dentistry, Rm. 406, 1919 7th Ave. S., Birmingham, AL 35233 USA; 4 DentalMonitoring, Paris, France; 5 Department of Orthodontics (M/C 841), College of Dentistry, University of Illinois Chicago, 801 S. Paulina Street, RM 131, Chicago, IL 60612-7211 USA; 6 Department of Orthodontics, Faculty of Dentistry Tanta University, Tanta, Egypt

## Abstract

**Introduction:**

This study aimed to test the performance of DentalMonitoring’s (DM) artificial intelligence (AI) in detecting aligner tracking issues.

**Methods:**

This multicenter retrospective comparative study analyzed 3,323 assessments from 623 patients treated at multiple U.S. sites. DM’s AI performance was evaluated using a binary model (seated vs. unseated) and a three-level model (seated, slight unseat, noticeable unseat). AI outputs were compared against a reference standard established through independent case reviews performed by a panel of three U.S.-based orthodontic residents. Sensitivity, specificity, positive predictive value (PPV), and negative predictive value (NPV) were calculated.

**Results:**

For the binary comparison (seat vs. unseat), sensitivity was 93.2% and specificity 86.2%, with a PPV of 89.2% and an NPV of 94.4%. For the three-level comparison, the noticeable-unseat category demonstrated a sensitivity of 91.1% and a specificity of 90.5%, with a PPV of 66.1% and an NPV of 98.3%. The high NPV values across both models indicate that DM’s AI was particularly reliable in ruling out clinically meaningful unseat events. The lower PPV in the noticeable-unseat category reflects the low prevalence of noticeable unseats in the dataset.

**Conclusion:**

DM’s AI system demonstrated high sensitivity and negative predictive values in identifying unseat events and in differentiating noticeable from slight unseats within the positive subset. These results indicate that the model performed reliably within the parameters and dataset evaluated, particularly in minimizing false-negative assessments of clinically meaningful misfits. Further validation in independent cohorts and across broader clinical contexts is warranted to confirm generalizability.

## Introduction

With the ongoing advancements in clear aligner techniques, Clear Aligner Therapy (CAT) has been increasingly favored within the orthodontic community over traditional fixed appliances. CAT is attractive to both practitioners and patients due to its numerous benefits, including enhanced comfort, improved aesthetics, ease of oral hygiene, better maintenance of dental and periodontal health, and reduced chairside time [1–3]. Nevertheless, this gain in popularity has raised concerns regarding the efficiency of tooth movement, particularly in the older population. Regardless of the system used, the companies that promote the use of Clear Aligners (CA) make numerous claims about the effectiveness and predictability of their systems, despite the limited availability of high-quality evidence that substantiates those claims [4]. In fact, the literature shows that the clinical realization of the intended movements typically falls short of the prescribed and approved corrections [5–7]. In addition, the predictability of difficult-to-be-achieved tooth movement through CAT is often overestimated by practitioners with limited clinical experience [8]. Consequently, additional series of refinement trays are frequently required, resulting in extra financial and time commitments for both patients and providers [9, 10]. This discrepancy between simulated and actual outcomes can be attributed to three potential factors.

The first reason is insufficient daily wear time. A retrospective cohort study of 2,644 adults treated with clear aligners used an app-based questionnaire to track aligner-change dates and self-reported daily wear time [11]. Only 36% met recommended wear-time targets, and 25% showed poor compliance. Women were less compliant than men, and patients without prior orthodontic treatment adhered better.

Second, Patients may also switch to the next aligner before completing the intended movement. Wear-time recommendations have long been debated. Early CAT guidance suggested 2-week wear per tray [12]. Subsequent work proposed 10-day intervals [13]. In 2016, Invisalign^®^ (Align Technology) advised 7-day wear (“How Does Invisalign Work | Invisalign Braces,” n.d.). As a result, orthodontist instructions typically range from 7 to 14 days or more. Even though previous literature has been limited in addressing an appropriate aligner change interval, one randomized clinical study has directly evaluated the efficacy of tooth movement with different aligner wear protocols of 7, 10, and 14 days [9]. This study demonstrated the comparable accuracy of the 7-day and 14-day protocols, with the 7-day protocol offering shorter treatment times, and therefore, a viable option for clinical treatment; additionally, the study specifically recommended to follow the 14-day protocol for complex posterior movements or angular movements (i.e. torque, tip, rotation) due to a statistically significant difference, however, they demonstrated no clinically significant difference (Al-Nadawi et al. 2021).

Third, and linked to the two reasons mentioned above, the aligners may not fit properly. This lack of intimate contact between the aligner and the tooth compromises the necessary forces required for effective tooth movement. Given the long appointment intervals for clear aligner patients, fitting issues may go undetected for several months, worsening progressively over several sets of aligners [10]. Remote monitoring has been shown to improve compliance among patients treated with clear aligners. In an interrupted-time-series analysis using a mobile app with e-reminders, the proportion of poorly compliant aligner patients dropped from 24.5% to 9.3% [14]. In a separate retrospective study of 100 tele-orthodontic users, reported aligner wear increased from 14 h to 22 h per day [15]. 

As remote monitoring technologies expand, many of these systems increasingly incorporate artificial intelligence (AI) to automate image analysis and support clinical decision-making. AI-assisted remote monitoring and big-data predictive analytics are already being used in medicine to interpret patient-submitted images and detect early complications [16–18]. In dentistry, deep learning models have been applied to remotely evaluate caries and early signs of oral cancer using smartphones [19, 20]. In orthodontics, emerging AI systems like DM enable patients to capture high-quality images for remote clinical assessment, track tooth movement between visits, and generate 3D models with accuracy comparable to those produced by intraoral scanners [21, 22]. One study evaluated DM’s ability to detect aligner tracking issues and found that it accurately identifies noticeable aligner unseats, with an overall accuracy of 93.1% [23]. 

Currently, there is a lack of independent, rigorous validation for AI-driven remote monitoring systems like DentalMonitoring (DM) in detecting aligner tracking issues. The primary aim of this study was to evaluate the sensitivity and specificity of DM’s AI algorithm in detecting aligner tracking issues, using a consensus of expert orthodontists as the reference standard.

## Materials and methods

### Study design & rationale

This was a multi-centric retrospective qualitative comparative study that included a dataset from 623 patients. The study was conducted in the scope of an FDA approval process, and the data were extracted from DM’s data pool.

This study was conducted in accordance with the ethical principles that have their origin in the Declaration of Helsinki. Applicable data protection regulations were also followed, namely Regulation (EU) 2017/745 and ISO 14155:2020. An exemption from the Western Institutional Review Board (WITH^®^) known as WCG IRB (WIRB-Copernicus Group IRB), was granted under 45 CFR § 46.104(d)(4). Because this study was a retrospective study the WCG IRP granted exemption for a Consent form (Human Ethics and Consent to Participate declarations: not applicable).

**Fig. 1:**
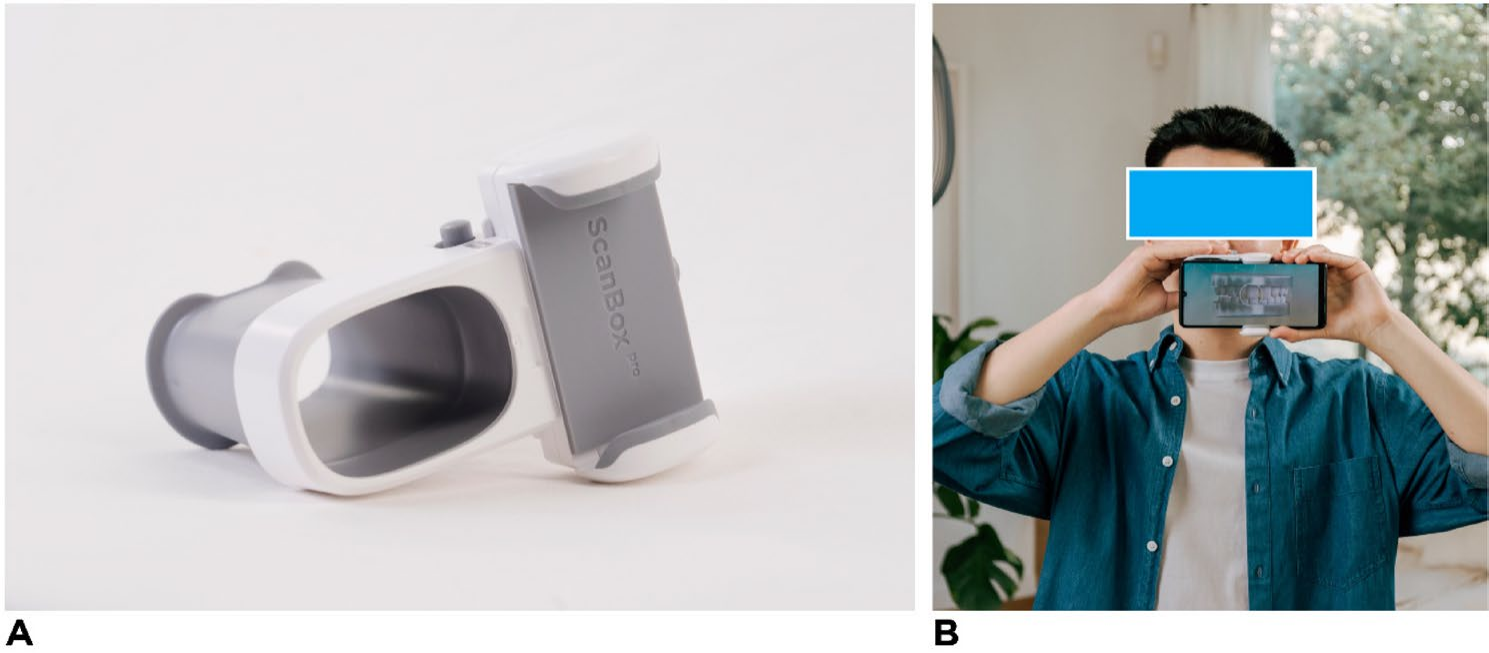
**A** The DM ScanBox Pro to support remote intraoral image acquisition by patients. **B** A patient performing a scan using the DM ScanBox Pro with a smartphone inserted, allowing image capture of occlusal and intraoral structures

The patient inclusion criteria included: Adolescents & adults of 13 years old and older and patients treated with aligners with at least one attachment. The patient exclusion criteria included: Patients with at least one primary tooth, patients ≤ 12 years old and non-US and non-European patients. With regard to the picture sets generated from the DM scans, the inclusion criteria were: De-identified DM picture sets, picture set acquired using the DM app (on a phone with at least Android 6 and up, or iOS 11 and up), a DM Cheek Retractor and a DM ScanBox (Fig. 1) and lastly a DM picture set with at least eight images processable by DM respecting the following reparation: At least three closed-view images (left, right, and front), at least three open-view images (left, right, and front), and two occlusal-view images (up and down).

The study aimed to evaluate DM’s performance in detecting aligner tracking issues based on predefined clinical categories and system outputs. Aligner tracking was assessed according to three classifications: satisfactory aligner tracking (seat), slight unseat, and noticeable unseat. A seat was defined as a satisfactory fit of the aligner with no visible unseats or damage. A slight unseat occurred when the aligner was not in close contact with the tooth, showing a minor gap of less than 1 mm between the incisal or occlusal edge and the aligner. A noticeable unseat referred to a poor aligner fit with a visibly greater gap exceeding 1 mm.

**Fig. 2:**
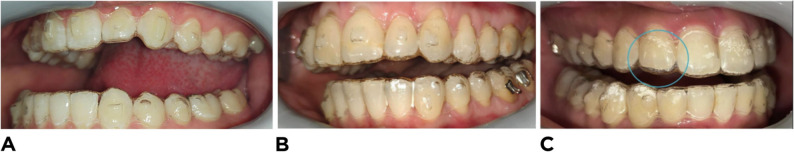
Examples of aligner seating conditions captured using the DM ScanBox and DM App, illustrating satisfactory aligner tracking (**A**), a slight unseat on UL2 (**B**), and a noticeable unseat on UR2 (**C**)

**Fig. 3:**
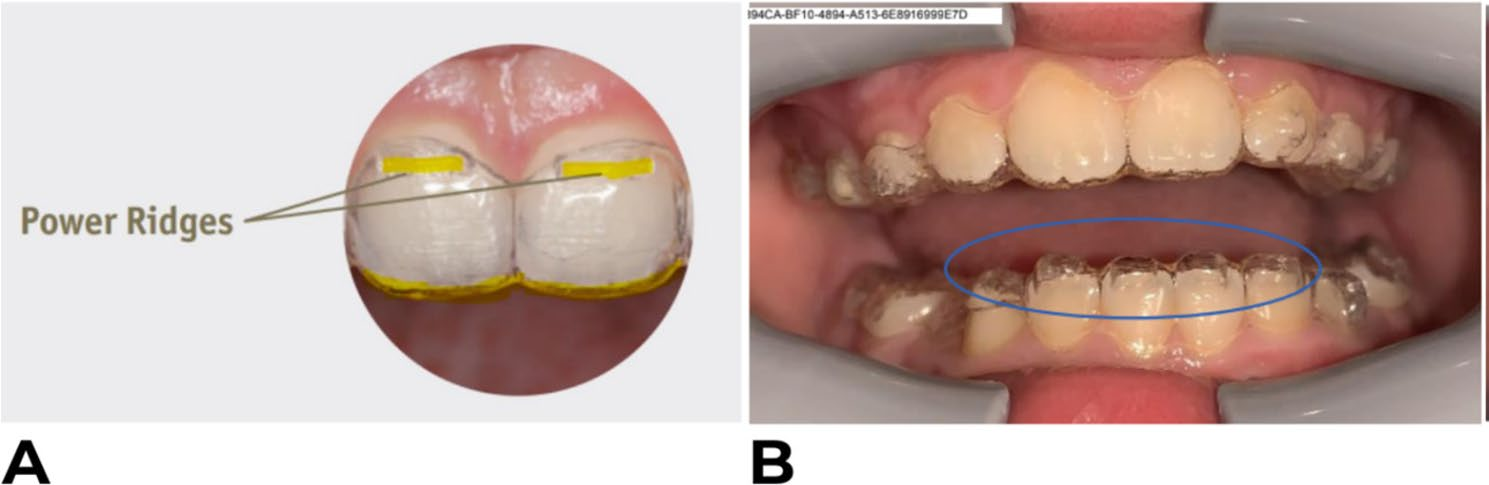
Examples of clear aligner features captured using the DM ScanBox and DM App, showing power ridges (**A**) and bite ramps (**B**)

These categories were evaluated under the clinical context of aligner and thermoformed retainer tracking. The possible outputs generated by the system for this parameter were: satisfactory tracking, slight unseat, noticeable unseat, cannot identify, and not applicable.Teeth presenting power ridges or bite ramps were excluded because these built-in aligner features can alter crown contours, and the standard DM workflow instructs users to exclude such teeth to avoid measurement noise. Although the DM algorithms are trained to recognize these features, that functionality was not assessed in this study. Examples of these outputs are shown in Figs. 2 and 3.

Prior to study initiation, DM’s Clinical Affairs team provided standardized training to all participating sites and expert panel members. The objective was to ensure consistent application of the study protocol, diagnostic definitions, and the use of the established reading scale, which has been widely used in radiology research studies [24]. To minimize potential bias, the Clinical Affairs team’s involvement was strictly limited to procedural training and troubleshooting prior to the formal initiation of the study. They were not involved in data selection, or data analysis during the study.

Following the initial instruction, a familiarization phase was conducted to validate investigators’ readiness to perform the assessments. During this phase, the investigators reviewed a set of ten representative mock cases, distinct from the study dataset, to practice labeling procedures, apply study group definitions, and complete data entry according to protocol. Each investigator used a dedicated computer with identical screen specifications, ensuring standardized assessment conditions.

**Fig. 4:**
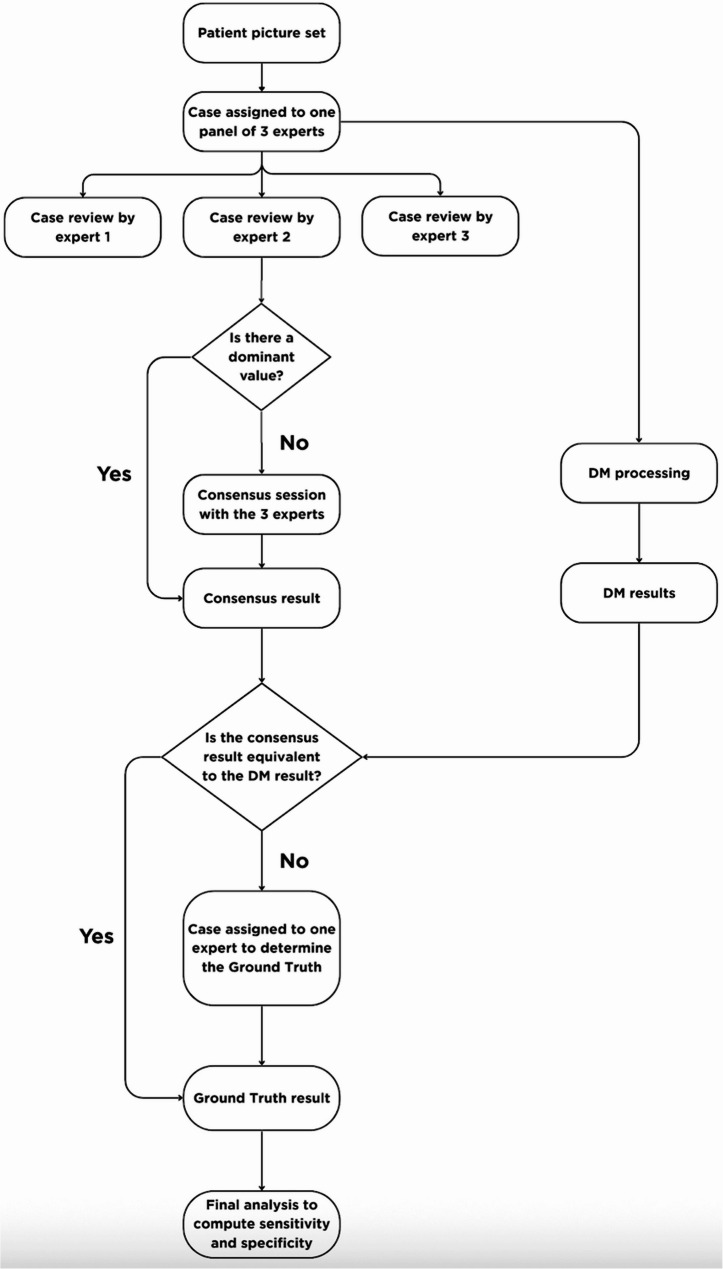
Workflow for establishing the Ground Truth, including independent expert review, consensus determination, DM result comparison, and external expert adjudication for discrepant cases

For each case, a DM intraoral picture set consisting of at least eight images from a single scan was processed by the DM software to generate an automated result. The same image set was independently reviewed by a panel of three experts who are US-based orthodontic residents. Each expert provided an assessment for every tooth and parameter, yielding three evaluations per item. These assessments were then examined to establish a panel consensus (Fig. 4). When all three experts agreed, that response was accepted as the final panel result. If two experts provided the same answer, the majority opinion was used. Cases with no agreement (three different answers) were discussed collectively until a consensus was reached.

The panel’s consensus result was subsequently compared with the DM-generated result. When both matched, the consensus was accepted as the Ground Truth. For cases in which the two results differed, an additional adjudication step was implemented: an external expert, who is an experienced board-certified orthodontist, reviewed the case to determine the final Ground Truth. The external reviewer was provided with detailed definitions and examples of the parameter under evaluation, and the complete intraoral image set.

The final Ground Truth dataset served as the Reference Standard for all statistical analyses.

### Statistical analysis

The sensitivity and specificity, with their 95% confidence intervals, were calculated using a Generalized Estimating Equations (GEE) model. This approach accounts for correlations between multiple teeth from the same patient, ensuring robust estimates. The model is particularly suited for this study, where each patient contributes multiple results due to the inclusion of several teeth.

To evaluate the diagnostic performance of DM, results were classified in comparison to the ground truth using a standard 2 × 2 contingency framework. A result was considered a true positive (TP) when the method correctly identified a positive case, and a true negative (TN) when it correctly identified a negative case. A false positive (FP) indicated an incorrect identification of a negative case as positive, while a false negative (FN) referred to a missed positive case.

Based on this classification, sensitivity was calculated as TP/(TP + FN), representing the method’s ability to correctly detect true positive findings. Specificity was calculated as TN/(FP + TN), reflecting the method’s accuracy in identifying true negatives. These metrics were used to quantify the reliability of the system in detecting clinical events.

Positive predictive value (PPV) and negative predictive value (NPV) were also calculated to quantify the probability that positive and negative DM findings corresponded to the ground truth. PPV was computed as TP/(TP + FP), indicating the proportion of positive detections that were truly positive. NPV was calculated as TN/(TN + FN), representing the proportion of negative detections that were truly negative. These values reflect the diagnostic performance of the system within the prevalence distribution of the study sample and provide complementary information on the clinical trustworthiness of positive and negative DM outputs.

## Results

The total number of results included in the final statistical analysis was 3,323 results from over 623 patients. The final comparisons and results for the two-level and three-level parameters are presented in Tables 1 and 2.

**Table 1:** Final comparison between DM outputs and ground truth for the two-level and three-level unseat classifications. The upper section (“Seat vs Unseat”) includes all evaluated teeth, with “Positive” representing either slight or noticeable unseat. The lower section (“Noticeable vs slight Unseat”) is a subset of the positive cases from the upper section and reports the differentiation between noticeable and slight unseats only within that positive subset

Seat vs. Unseat			
	Result	95% CI lower bound	95% CI upper bound
Sensitivity	93.2%	91.3%	94.7%
Specificity	86.2%	83.4%	88.6%
Noticeable vs. Slight Unseat			
	Result	95% CI lower bound	95% CI upper bound
Sensitivity	91.10%	85.90%	94.50%
Specificity	90.50%	87.80%	92.70%

**Table 2:** Final sensitivity and specificity results for the two-level and three-level unseat parameters. The upper section (“Seat vs Unseat”) reports performance across all evaluated teeth, with unseats defined as either slight or noticeable. The lower section (“Noticeable vs slight Unseat”) corresponds to the subset of teeth classified as unseated in the two-level analysis and presents the model’s ability to distinguish noticeable from slight unseats within that subset

Seat vs. Unseat				
		Ground truth		
		Positive (noticeable or slight)	Negative	Total
DM results	Positive (noticeable or slight)	1360	165	1525
	Negative	101	1697	1798
	Total	1461	1862	3323
Noticeable vs. Slight Unseat				
		Ground truth		
		Noticeable	Slight	Total
DM result	Noticeable	193	99	292
	Slight	18	1050	1068
	Total	211	1149	1360

For the two-level classification task (Seat vs. Unseat), the model demonstrated a sensitivity of 93.2% (95% CI: 91.3%–94.7%) and a specificity of 86.2% (95% CI: 83.4%–88.6%). Based on the observed prevalence within the study sample, the corresponding positive predictive value (PPV) was 89.2%, while the negative predictive value (NPV) reached 94.4%.

For the three-level comparison between noticeable and slight unseat, the model achieved a sensitivity of 91.1% (95% CI: 85.9%–94.5%) and a specificity of 90.5% (95% CI: 87.8%–92.7%). In this subset, the PPV was 66.1%, and the NPV was 98.3%, reflecting the lower prevalence of noticeable unseats within this dataset.

Finally, to make sure that the study covers all the teeth of a patient’s dentition, it was verified that each tooth number represented at least 1% of the total number analyzed. This distribution is presented in Table 3.

**Table 3:** Distribution of individual teeth included in the analysis, showing the percentage of total assessments contributed by each tooth

Tooth #	Frequency	Tooth #	Frequency
11	5.5%	31	6.4%
12	6.0%	32	5.6%
13	3.1%	33	4.0%
14	2.7%	34	3.3%
15	2.9%	35	2.8%
16	2.2%	36	2.3%
17	2.1%	37	2.0%
21	4.8%	41	6.3%
22	6.2%	42	5.5%
23	3.2%	43	3.3%
24	2.2%	44	3.6%
25	2.6%	45	2.5%
26	2.6%	46	1.9%
27	1.9%	47	2.3%

Descriptive statistics for all recruited subjects who completed the study were performed for gender, age (years), and location (Table 4).

**Table 4:** Distribution of individual teeth included in the analysis, showing the percentage of total assessments contributed by each tooth

Characteristic	# (%)
Age group	
13; 22 years of age	291 (46.7%)
≥ 22 years of age	332 (53.3%)
Location	
Central	286 (45.9%)
East Coast	192 (30.8%)
West Coast	17 (2.7%)
Out of US	8 (1.3%)
Western	85 (13.6%)
US - Other	35 (5.6%)

## Discussion

This study aimed to evaluate the performance of Artificial Intelligence–Driven Remote Monitoring (AIDRM) in detecting aligner tracking issues using two classification schemes: a binary model (seat vs. unseat) and a three-level model (seated, slight unseat, and noticeable unseat). The study cohort included patients from multiple geographic regions across the United States, spanning different age groups and treated with several commercial aligner brands. To minimize positional bias and reflect real-world clinical conditions, all teeth were included in the analysis.

The results demonstrated that DM achieved high diagnostic performance across both classification tasks. In the binary comparison, sensitivity was 93.2% and specificity 86.2%, with a PPV of 89.2% and an NPV of 94.4%. These values indicate that DM was highly reliable in identifying true unseat events and particularly effective at ruling them out. In the three-level model, sensitivity and specificity remained high (91.1% and 90.5%, respectively). The NPV was exceptionally high at 98.3%, reflecting the system’s strong ability to exclude noticeable misfits. The PPV for noticeable unseat was lower (66.1%), which can be explained by the low prevalence of noticeable unseats within the sample and the known dependence of PPV on event prevalence. Together, these findings suggest that DM functions as a highly sensitive, safety-oriented system that minimizes false negatives while preserving strong discriminative ability across different levels of aligner misfit severity.

When compared with the study by Tahir A., meaningful parallels and distinctions emerge [25]. Tahir reported overall detection accuracies exceeding 90% for clear-aligner tracking parameters, but noted reduced sensitivity for subtle findings such as slight unseat (76.8%). This pattern aligns with our multi-level results, in which the noticeable-unseat category showed a lower PPV due to the low prevalence of noticeable cases, while still maintaining high sensitivity and an excellent NPV. In contrast to Tahir’s study, which evaluated a more uniform set of aligner systems, the present study included multiple commercial aligner brands, introducing greater variability and enhancing the generalizability of the findings. Because this was a retrospective study based on randomly selected records, specific information on which aligner brands were used for each patient was not available in the extracted dataset and therefore could not be reported. Although all teeth were included in the analysis to minimize positional bias, tooth-specific performance metrics (e.g., sensitivity or specificity per tooth type) were not computed in this study. Given the clinical relevance of tooth-level variability and prior indications from Tahir’s work that certain teeth may be more challenging to detect, this represents an area for future investigation [18]. 

The literature indicates that the predictability of OTM with CA is influenced by numerous factors, including the clinician’s experience [8], the nature of tooth movement [5, 26, 27], patient compliance and aligner wear time, the interval between aligner changes, the amount of programmed tooth movement [28], and even the type of aligner used. Studies have shown that with in-house aligners, there is a significant discrepancy between achieved and predicted tooth movements [29]. Additionally, other biological factors such as age, sex, systemic diseases, and medications also play a role [30]. 

These limitations in expressing predicted tooth movements ultimately pose a challenge in achieving clinically acceptable results and highlight the need for careful consideration and planning in CAT protocols. Given the complexity and variability of these factors, some of which are beyond the clinician’s control, advancements in artificial intelligence and remote monitoring present a significant opportunity for developing individualized aligner change protocols that account for the patient’s biological response [31]. A recent study conducted at Harvard University evaluated the ability of DM to personalize aligner wear time and its impact on treatment duration. It found that patients using DM progressed through aligners more quickly than the standard 7- or 14-day protocols [32]. For the four groups monitored with DM, the average frequency of aligner changes ranged from 4.315 to 5.370 days per tray. The study’s author emphasized that this does not mean every patient can change trays every 4 to 5 days; for instance, one patient took 25 days to receive a “GO” on the 9th aligner. Consequently, this personalization of the treatment schedule not only enhances efficiency but also maintains treatment quality. As found in Tahir A. et al. study, the DM group achieved similar treatment outcomes compared to conventional treatment groups, based on the American Board of Orthodontics (ABO) Discrepancy Index (DI) and Objective Grading System (OGS).

This study has several limitations that should be considered when interpreting the findings. First, the retrospective design may introduce selection bias and limit control over confounding variables. Second, the study was funded by DentalMonitoring as part of an FDA submission, and some investigators were compensated for their time, which introduces a potential conflict of interest. Third, although all teeth were included to minimize positional bias, tooth-specific performance metrics such as sensitivity or specificity by tooth type were not computed and should be evaluated in future work. Fourth, the consistency of the AI’s performance across repeated assessments over the course of treatment was not investigated and longitudinal analyses are needed to confirm consistency and reliability of the findings.

As AIDRM gains in popularity, future research endeavors should meticulously assess the systems’ reliability while examining the ramifications of this innovative approach on patient care quality, experience, and treatment efficiency.

## Conclusion

The findings of this study indicate that DM’s AI demonstrates strong diagnostic performance in detecting aligner tracking issues across both binary and three-level classifications. The high sensitivity and high NPV observed in this analysis suggest that the system is particularly effective at identifying and ruling out clinically meaningful unseat events. By providing timely notifications following patient scans, DM supports clinicians in making prompt, informed decisions during treatment.

Given the absence of evidence supporting a universal fixed interval for clear aligner changes, AI-driven remote monitoring offers a valuable opportunity to develop individualized aligner progression protocols. Such an approach has the potential to better reflect each patient’s biological response and may contribute to more efficient and adaptive clear aligner therapy in future clinical applications.

## Data Availability

All data generated during this study are included in this published article and its information files.

## Abbreviations

CA: Clear Aligners
CAT: Clear Aligner Therapy
OTM: Orthodontic Tooth Movement
AIDRM: Artificial Intelligence Driven Remote Monitoring
